# Supplementary material for: CB2 cannabinoid receptor-specific therapeutic antibody agonists for treatment of chemotherapy-induced peripheral neuropathy
Source: bioRxiv. 2025 Dec 1:2025.11.26.690750. Preprint. [Version 1] doi: 10.1101/2025.11.26.690750 (PMC12694584; doi:10.1101/2025.11.26.690750)
Supplement: 1 [file NIHPP2025.11.26.690750V1-supplement-1.pdf]

bioRxiv preprint doi: https://doi.org/10.1101/2025.11.26.690750; this version posted December 1, 2025. The copyright holder for this preprint (which was not certified by peer review) is the author/funder, who has granted bioRxiv a license to display the preprint in perpetuity. It is made available under aCC-BY 4.0 International license.

Supplemental Figure Legends

Supplemental Figure 1. AB110 and paclitaxel produce additive effects in 4T1 cells to reduce murine mammary carcinoma cell line viability. (**A**) Dose-response for the effect of paclitaxel in 4T1 cell viability (EC50=21.5 nM). (**B**) Dose-response curve for the effect of AB110 in 4T1 cell viability (EC50>50 µM). (**C**) Paclitaxel dose response shift observed in the presence of increasing concentrations of AB110. (**D**) AB110 dose response shift observed in the presence of increasing concentrations of paclitaxel in 4T1 cell viability. (**E–G**) The 3-dimensional landscape of the dose matrix is represented on a color scale, where blue reflects evidence of synergy and red reflects evidence of antagonism. The landscape of the dose matrix of combination responses for AB110 and paclitaxel based on the (**E**) Bliss model, (**F**) Highest Single Agent (HSA) model and (**G**) Loewe model. Each model supports additive effects of the combination in reducing 4T1 tumor cell line viability. Cell viability is plotted as % control. (n = 3 experiments).

Supplemental Figure 2. AB100 produces slight antagonism or additive effects with paclitaxel in 4T1 cells to reduce murine mammary carcinoma cell line viability. (**A**) Dose-response for the effect of paclitaxel on 4T1 cell viability (EC50=31.9 nM). (**B**) Dose-response curve for the effect of AB100 in 4T1 cell viability (EC95=4.451 µM). (**C**) Paclitaxel dose response shift observed in the presence of increasing concentrations of AB100 in 4T1 cell viability. (**D**) AB100 dose response shift observed in the presence of increasing concentrations of paclitaxel in tumor cell viability (4T1). (**E–G**) The 3-dimensional landscape of the dose matrix is represented on a color scale, where blue reflects evidence of synergy and red reflects evidence of antagonism. The landscape of the dose matrix of combination responses for AB100 and paclitaxel based on the (**E**) Bliss model, (**F**) Highest Single Agent (HSA) model and (**G**) Loewe model. Each model supports either modest antagonism or additive effect of the combination in reducing 4T1 tumor cell line viability. Cell viability is plotted as % control. (n = 3 experiments).

bioRxiv preprint doi: https://doi.org/10.1101/2025.11.26.690750; this version posted December 1, 2025. The copyright holder for this preprint (which was not certified by peer review) is the author/funder, who has granted bioRxiv a license to display the preprint in perpetuity. It is made available under aCC-BY 4.0 International license.

Supplemental Figure 3. Additive effects of AB110 and paclitaxel on nontumor HEK293 cells. (**A**) Dose-response for the effect of paclitaxel on viability of HEK293 cells (EC50=123 nM). (**B**) Dose-response curve for the effect of AB110 on viability of HEK293 cells (EC50>50 µM). (**C**) Paclitaxel dose response shift observed in the presence of increasing concentrations of AB110 in non-tumor cell viability (HEK293). (**D**) AB100 dose response shift observed in the presence of increasing concentrations of paclitaxel in non-tumor cell viability. (**E–G**) The 3-dimensional landscape of the dose matrix is represented on a color scale, where blue reflects evidence of synergy and red reflects evidence of antagonism. The landscape of the dose matrix combination responses for AB110 and paclitaxel based on the (**E**) Bliss model, (**F**) Highest Single Agent (HSA) model and (**G**) Loewe model. Cell viability is plotted as % control (n = 4 experiments).

Supplemental Figure 4. Additive effects of AB100 and paclitaxel on the viability of non-tumor HEK293 cells. (**A**) Dose-response curve for the effect of paclitaxel on viability of HEK293 cells (EC50>500 nM). (**B**) Dose-response for the effect of AB100 on viability of HEK293 cells (EC95=17 nM). (**C**) Paclitaxel dose response shift observed in the presence of increasing concentrations of AB100. (**D**) AB100 dose response shift observed in the presence of increasing concentrations of paclitaxel. (**E–G**) The 3-dimensional landscape of the dose matrix is represented on a color scale, where blue reflects evidence of synergy and red reflects evidence of antagonism. The landscape of the dose matrix combination responses for AB100 and paclitaxel based on the (**E**) Bliss model, (**F**) Highest Single Agent (HSA) model and (**G**) Loewe model. Cell viability is plotted as % control (n = 4 experiments).

bioRxiv preprint doi: https://doi.org/10.1101/2025.11.26.690750; this version posted December 1, 2025. The copyright holder for this preprint (which was not certified by peer review) is the author/funder, who has granted bioRxiv a license to display the preprint in perpetuity. It is made available under aCC-BY 4.0 International license.

Supplemental Figure 1

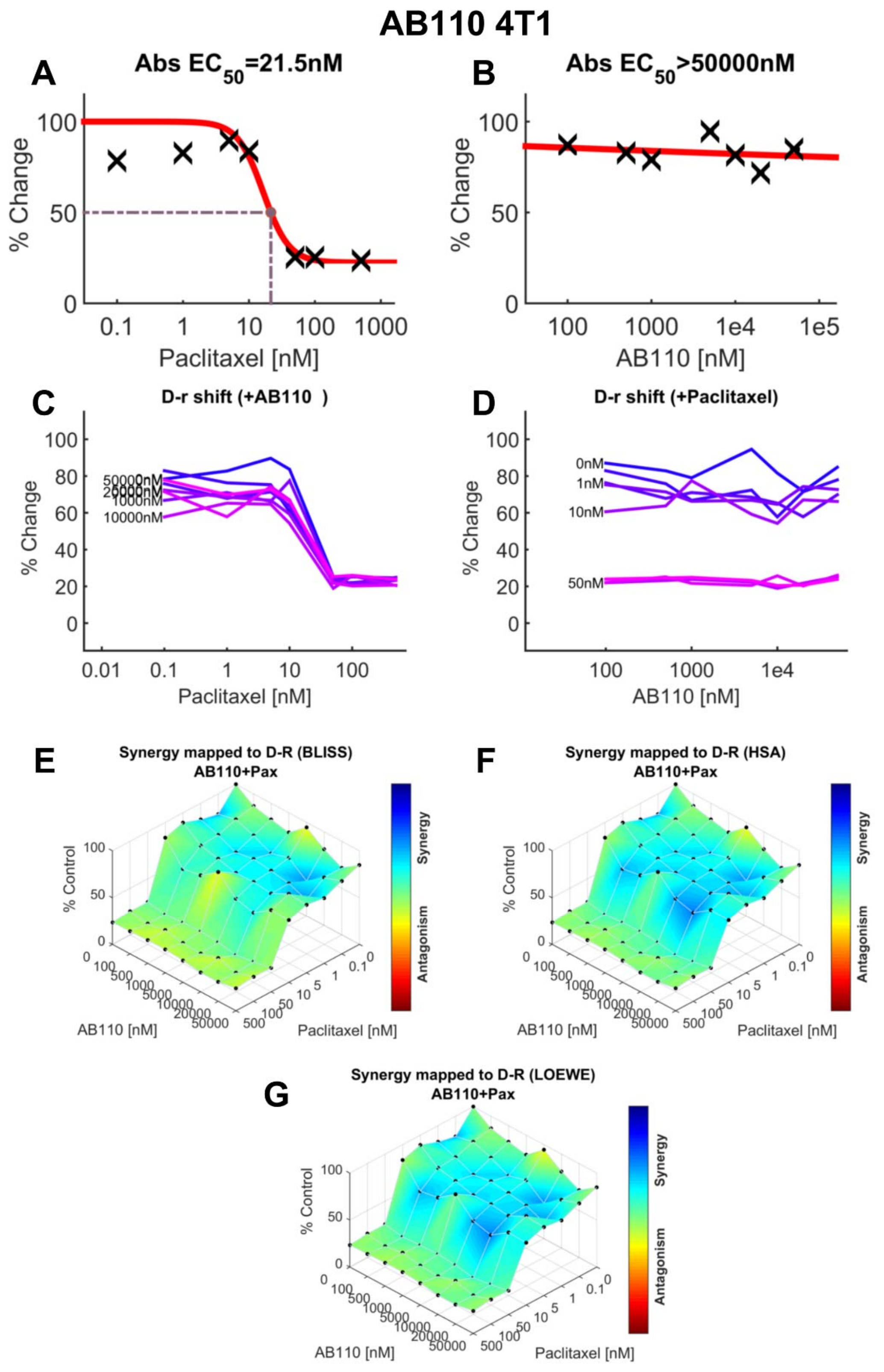

bioRxiv preprint doi: https://doi.org/10.1101/2025.11.26.690750; this version posted December 1, 2025. The copyright holder for this preprint (which was not certified by peer review) is the author/funder, who has granted bioRxiv a license to display the preprint in perpetuity. It is made available under aCC-BY 4.0 International license.

Supplemental Figure 2

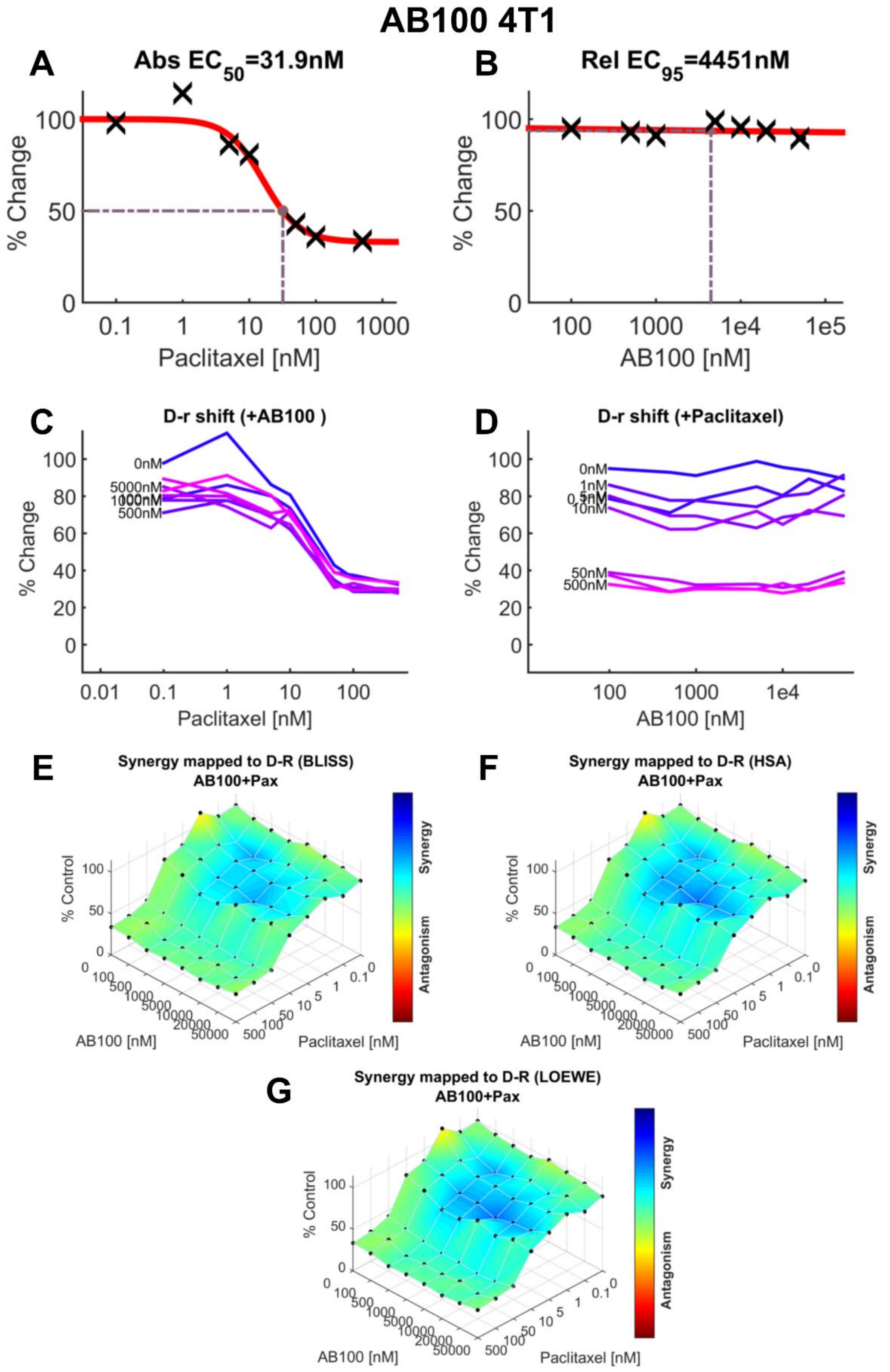

bioRxiv preprint doi: https://doi.org/10.1101/2025.11.26.690750; this version posted December 1, 2025. The copyright holder for this preprint (which was not certified by peer review) is the author/funder, who has granted bioRxiv a license to display the preprint in perpetuity. It is made available under aCC-BY 4.0 International license.

Supplemental Figure 3

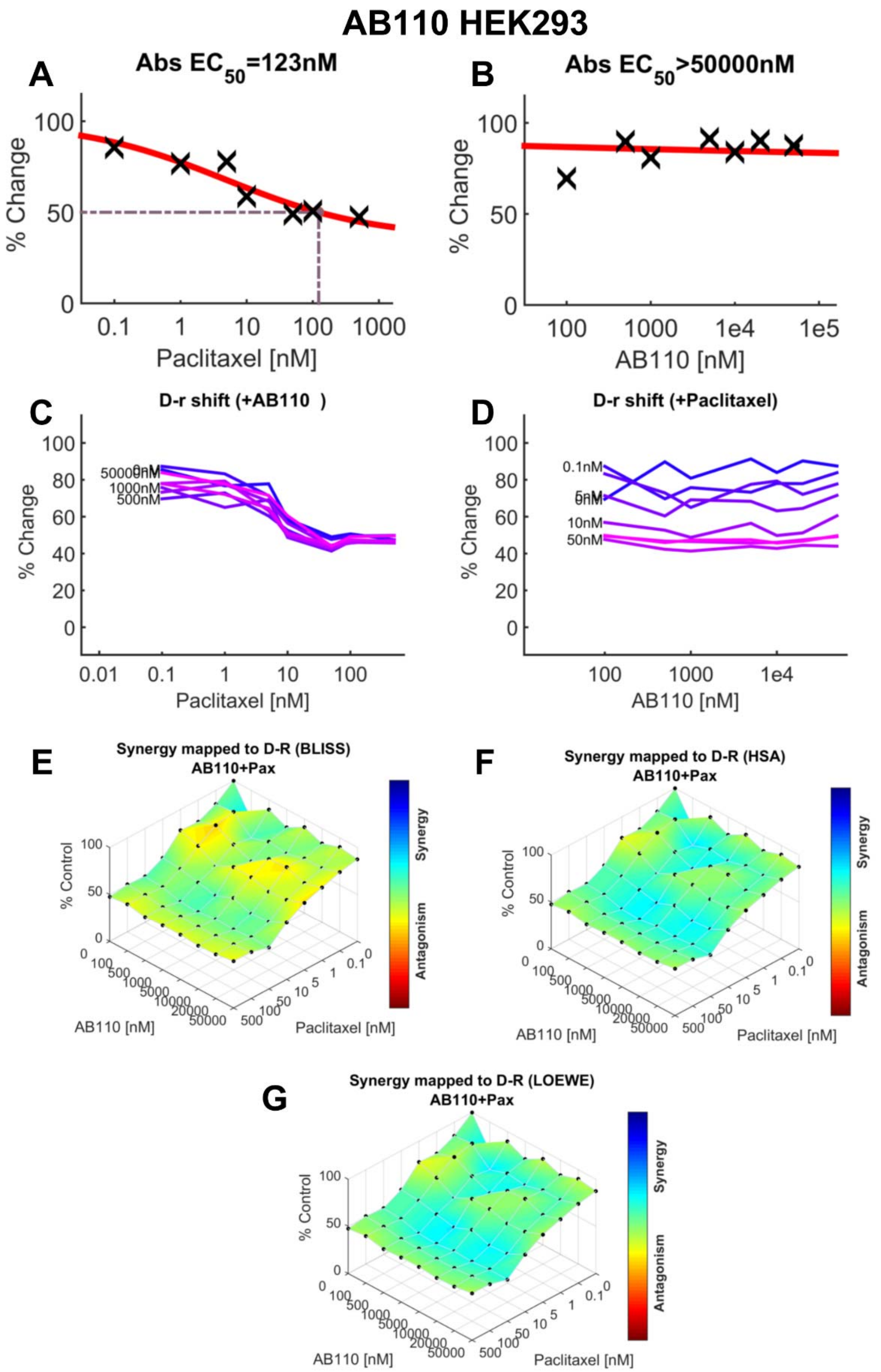

bioRxiv preprint doi: https://doi.org/10.1101/2025.11.26.690750; this version posted December 1, 2025. The copyright holder for this preprint (which was not certified by peer review) is the author/funder, who has granted bioRxiv a license to display the preprint in perpetuity. It is made available under aCC-BY 4.0 International license.

Supplemental Figure 4

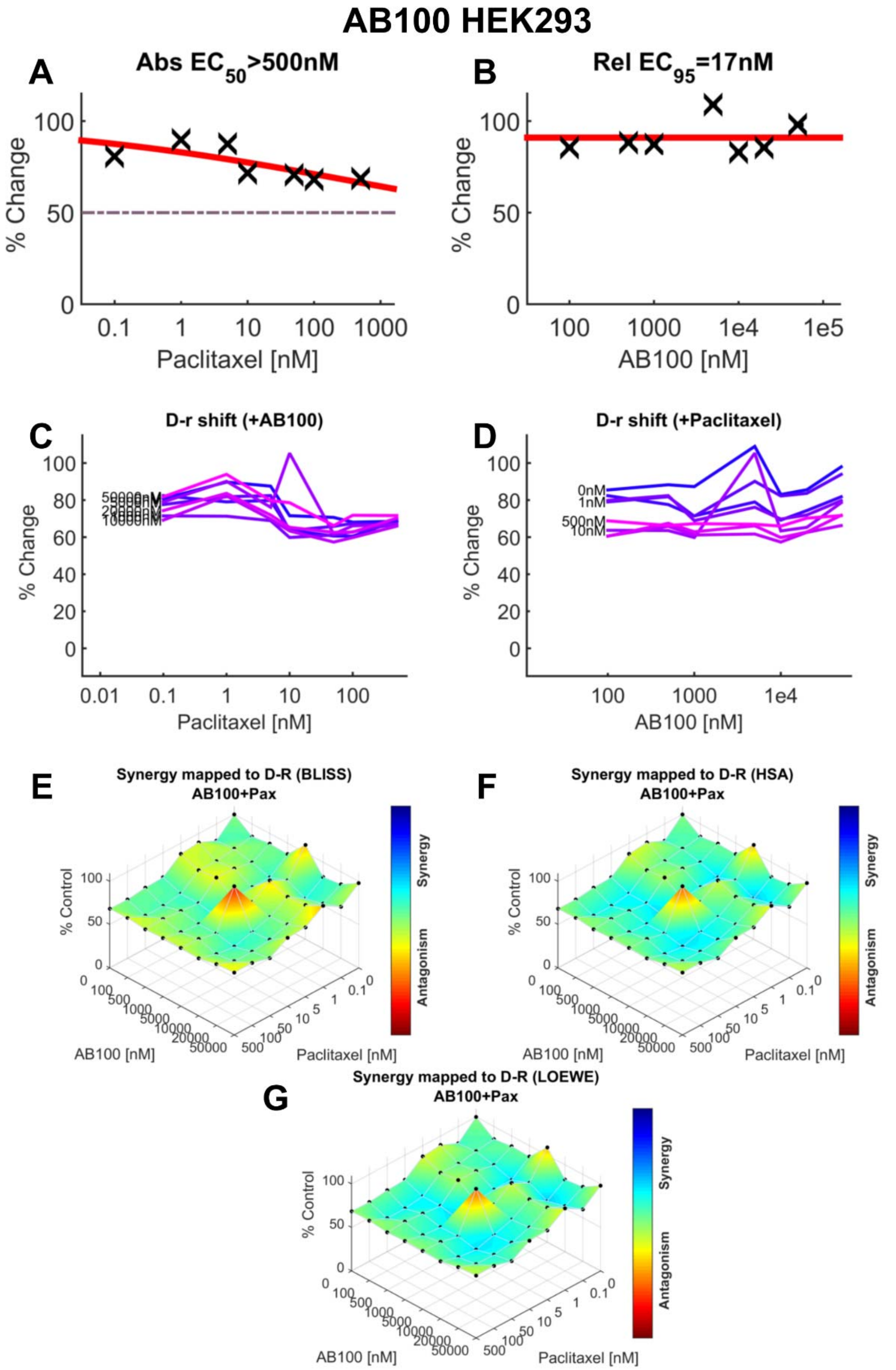

bioRxiv preprint doi: https://doi.org/10.1101/2025.11.26.690750; this version posted December 1, 2025. The copyright holder for this preprint (which was not certified by peer review) is the author/funder, who has granted bioRxiv a license to display the preprint in perpetuity. It is made available under aCC-BY 4.0 International license.

Supplemental Table 1. Primers used in real-time PCR

No	Target Gene	Primer sequence
1	ARG1-F<br>ARG1-R	5□- CTCCAAGCCAAAGTCCTTAGAG-3□<br>5□- AGGAGCTGTCATTAGGGACA-3□
2	IL1-β-F<br>IL1-β-R	5□-GCAACTGTTCCTGAACTCAACT-3□<br>5□-ATCTTTTGGGGTCCGTCAACT-3□
3	IL6-F<br>IL6-R	5□- CACATGTTCTCTGGGAAATCGTGGA-3□<br>5□- TCTCTCTGAAGGACTCTGGCTTTGT-3□
4	NOS2-F<br>NOS2-R	5□- GTTCTCAGCCCAACAATACAAGA-3□<br>5□- GTGGACGGGTCGATGTCAC-3□
5	TNF-α-F<br>TNF-α-R	5□- CCCTCACACTCAGATCATCTTCT-3□<br>5□- GCTACGACGTGGGCTACAG-3□
6	MRC2-F<br>MRC2-R	5□- TCCTACAAATACACGCTGGTGGCGATA-3′<br>5□- GCAGTTCCCTTTTAAATGCAAATCA-3′
7	MGL1-F<br>MGL1-R	5□- CTTGGTCCCAGATCCGTATC-3′<br>5□- ATGTCATGACTCAGGATC-3′
8	CLEC7A-F<br>CLEC7A-R	5′- CCAGCTAGGTGCTCATCTACTG -3′<br>5′- CCTTCACTCTGATTGCGGGAAAG -3′
9	CCL4-F<br>CCL4-R	5’-AACACCATGAAGCTCTGCGT-3’<br>5’-AGAAACAGCAGGAAGTGGGA-3’
10	GAPDH-F<br>GAPDH-R	5□-AGGTCGGTGTGAACGGATTTG-3□<br>5□-TGTAGACCATGTAGTTGAGGTCA-3□

bioRxiv preprint doi: https://doi.org/10.1101/2025.11.26.690750; this version posted December 1, 2025. The copyright holder for this preprint (which was not certified by peer review) is the author/funder, who has granted bioRxiv a license to display the preprint in perpetuity. It is made available under aCC-BY 4.0 International license.

Supplemental Table 2. Efficacy and potency profiles of CB2 antibody agonists

Antibody	Figure	Assay	EC50 (nM)	Emax (%)
HU308	1A	Gi-cAMP	3.88	100
AB110	1A	Gi-cAMP	3.68	73.06
AB120	1A	Gi-cAMP	11.2	93.56
AB100	1A	Gi-cAMP	2.13	19.7
HU308	1B	Antagonist - Gi-cAMP	44.90	100
AB110	1B	Antagonist - Gi-cAMP	45.60	98.77
AB120	1B	Antagonist - Gi-cAMP	45.22	83.53
AB100	1B	Antagonist - Gi-cAMP	N/A	N/A
HU308	1C	β-Arrestin-2	100.6	100
AB110	1C	β-Arrestin-2	372.6	71.82
AB120	1C	β-Arrestin-2	213.0	45.35
AB100	1C	β-Arrestin-2	7.19	8.84

bioRxiv preprint doi: https://doi.org/10.1101/2025.11.26.690750; this version posted December 1, 2025. The copyright holder for this preprint (which was not certified by peer review) is the author/funder, who has granted bioRxiv a license to display the preprint in perpetuity. It is made available under aCC-BY 4.0 International license.

Supplemental Table 3. Synergy score to quantify degree of synergy for paclitaxel and CB2-specific antibody agonists

Cell Line	CB2 Antibody	Model	Synergy Score ± SEM	Most Synergistic Area Score
4T1	AB100	Bliss	6.68 ± 6.38	15.01
		HSA	8.66 ± 6.38	16.23
		Loewe	-15.22 ± 6.38	5.57
	AB110	Bliss	1.35 ± 3.31	10.79
		HSA	8.23 ± 3.31	18.86
		Loewe	8.85 ± 3.31	19.21
	AB120	Bliss	-7.5 ± 5.14	-2.67
		HSA	0.13 ± 5.14	6.49
		Loewe	N/A*	N/A*
HEK293	AB100	Bliss	1.79 ± 4.86	6.89
		HSA	5.91 ± 4.86	11.23
		Loewe	-2.19 ± 4.86	5.69
	AB110	Bliss	-4.95 ± 5.09	1.02
		HSA	3.44 ± 5.09	8.51
		Loewe	-8.89 ± 5.09	1.42
	AB120	Bliss	-0.93 ± 5.11	5.39
		HSA	7.74 ± 5.11	14.95
		Loewe	4.41 ± 5.11	12.92

Scores higher than 10 are likely synergistic, a score between −10 and 10 suggests that effects are likely additive, and scores less than −10 generally suggest evidence for antagonism. *Loewe model cannot calculate the synergy score with this combination data set.
